# Timing of Histologic Progression from Chorio-Deciduitis to Chorio-Deciduo-Amnionitis in the Setting of Preterm Labor and Preterm Premature Rupture of Membranes with Sterile Amniotic Fluid

**DOI:** 10.1371/journal.pone.0143023

**Published:** 2015-11-17

**Authors:** Chan-Wook Park, Joong Shin Park, Errol R. Norwitz, Kyung Chul Moon, Jong Kwan Jun, Bo Hyun Yoon

**Affiliations:** 1 Department of Obstetrics and Gynecology, Seoul National University College of Medicine, Seoul, Korea; 2 Department of Obstetrics and Gynecology, Tufts University School of Medicine, Boston, United States of America; 3 Department of Pathology, Seoul National University College of Medicine, Seoul, Korea; Tokyo Medical and Dental University, JAPAN

## Abstract

**Background:**

Histologic chorio-deciduitis and chorio-deciduo-amnionitis (amnionitis) in extra-placental membranes are known to represent the early and advanced stages of ascending intra-uterine infection. However, there are no data in humans about the time required for chorio-deciduitis to develop and for chorio-deciduitis without amnionitis to progress to chorio-deciduitis with amnionitis, and the effect of prolongation of pregnancy on the development of chorio-deciduitis and amnionitis in patients with preterm labor and intact membranes (PTL) and preterm premature rupture of membranes (preterm-PROM). We examined these issues in this study.

**Methods:**

The study population consisted of 289 women who delivered preterm (133 cases with PTL, and 156 cases with preterm-PROM) and who had sterile amniotic fluid (AF) defined as a negative AF culture and the absence of inflammation as evidenced by a matrix metalloproteinase-8 (MMP-8) level <23 ng/ml. We examined the association between amniocentesis-to-delivery interval and inflammatory status in the extra-placental membranes (i.e., inflammation-free extra-placental membranes, choroi-deciduitis only, and chorio-deciduitis with amnionitis) in patients with PTL and preterm-PROM.

**Results:**

Amniocentesis-to-delivery interval was longer in cases of chorio-deciduitis with amnionitis than in cases of chorio-deciduitis only in both PTL (median [interquartile-range (IQR)]; 645.4 [319.5] vs. 113.9 [526.9] hours; P = 0.005) and preterm-PROM (131.3 [135.4] vs. 95.2 [140.5] hours; P<0.05). Amniocentesis-to-delivery interval was an independent predictor of the development of both chorio-deciduitis and amnionitis after correction for confounding variables such as gestational age at delivery in the setting of PTL, but not preterm-PROM.

**Conclusions:**

These data confirm for the first time that, in cases of both PTL and preterm-PROM with sterile AF, more time is required to develop chorio-deciduitis with amnionitis than chorio-deciduitis alone in extra-placental membranes. Moreover, prolongation of pregnancy is an independent predictor of the development of both chorio-deciduitis and amnionitis in cases of PTL with sterile AF.

## Introduction

Ascending intra-uterine infection is the most common cause of spontaneous preterm birth [1–3]. It is hypothesized that infectious organisms from vagina gain access to the uterus through the cervix and then proceed to invade the chorio-decidua and finally the amnion in the extra-placental membranes [1–3]. Indeed, levels of inflammatory biomarkers in the amniotic fluid (AF) are increased in the presence of histologic chorio-deciduitis compared with inflammation-free placentas [4], and are increased even further in the presence of amnionitis compared with chorio-deciduitis alone [5]. However, there is no data in humans about the time required for ascending intra-uterine infection to invade the extra-placental membranes leading to histologic chorio-deciduitis and amnionitis in the setting of preterm labor and intact membranes (PTL) and preterm premature rupture of membranes (preterm-PROM). We hypothesized that more time would be required to develop histologic chorio-deciduitis with amnionitis than chorio-deciduitis without amnionitis in cases of both PTL and preterm-PROM with sterile AF, and prolongation of pregnancy would be an independent risk factor of chorio-deciduitis and amnionitis in the setting of PTL and preterm-PROM with sterile AF.

## Methods

### Study design and data collection

A retrospective cohort study was performed of 289 patients who delivered preterm (defined as delivery prior to 37 weeks of gestation) due to PTL (133 cases) or preterm-PROM (156 cases) from the year 1993 to 2007. Patients were eligible for inclusion in the analysis if they presented with a singleton pregnancy and PTL or preterm-PROM and also had: (1) an amniocentesis between 23.5 weeks and 36.0 weeks of gestation; (2) “sterile” AF as evidenced by a negative AF culture and the absence of AF inflammation defined as an AF matrix metalloproteinase-8 (MMP-8) level <23 ng/mL; and (3) documented results of placental pathology. On histologic examination of the placenta and extra-placental fetal membranes, inflammation was examined in choriodecidua, amnion, umbilical-cord, and chorionic-plate. The association between histologic findings and amniocentesis-to-delivery interval was then examined. Patients were divided into 3 groups according to the amniocentesis-to-delivery interval (≤2 days, 2–7 days, and >7 days). Transabdominal amniocentesis and placental pathology are routinely offered to patients admitted with a diagnosis of PTL or preterm-PROM at our institution. PTL was defined as the presence of regular phasic uterine contractions with a frequency of at least two contractions every 10 minutes and associated cervical change. Rupture of membranes was diagnosed at the time of sterile speculum examination by confirming the presence of pooling of AF in the vagina, a positive nitrazine pH test, and a positive ferning test result as previously reported [6, 7]. AF was routinely analyzed for intraamniotic infection and inflammation. We obtained a written informed consent in all subjects included in this study. The collection or use of these samples and information for research purposes were approved by the Institutional Review Board of Seoul National University Hospital. The Institutional Review Board of Seoul National University Hospital specifically approved this study.

The demographic and clinical characteristics of mothers and their neonates were abstracted from the medical records. Data abstracted included maternal age, parity, gestational age (GA) at amniocentesis, cervical dilatation at amniocentesis, GA at delivery, vaginal delivery or Cesarean section after trial of labor, birth weight, gender of newborn, 1 min and 5 min Apgar scores, umbilical cord arterial pH at birth (if collected), antenatal use of antibiotics, corticosteroids and tocolytic agents. Clinical chorioamnionitis was diagnosed when maternal temperature was elevated to 37.8°C and ≥ 2 of the following criteria were present: uterine tenderness, malodorous vaginal discharge, maternal leukocytosis (>15,000 cells/mm^3^), maternal tachycardia (>100 beats/min), and fetal tachycardia (>160 beats/min).

### Placental pathologic examination

Placental tissue samples included choriodecidua, amnion, umbilical-cord, and chorionic-plate. Samples were fixed in 10% neutral buffered formalin and embedded in paraffin. Hematoxylin / eosin staining was performed in the sections of prepared tissue-blocks. Clinical information was not disclosed to pathologists. Chorio-deciduitis, amnionitis, funisitis and chorionic plate inflammation were diagnosed according to previously published criteria [8], which included the following: (1) for chorio-deciduitis, at least one focus of more than five neutrophils in the choriodecidua; (2) for amnionitis, at least one focus of more than five neutrophils in the amnion; (3) for funisitis, neutrophil infiltration into the umbilical vessel walls or Wharton’s jelly; and (4) for chorionic-plate inflammation, more than one focus of at least ten neutrophilic-collections or diffuse inflammation in subchorionic-fibrin, or diffuse / dense inflammation, neutrophilic-infiltration into connective-tissue of placental-plate, or placental-vasculitis. For the purposes of the analysis, the histologic inflammatory status of the extra-placental membranes was classified into three groups: inflammation-free extra-placental membranes, chorio-deciduitis only, or chorio-deciduitis with amnionitis.

### Amniotic fluid testing

AF was cultured for aerobic / anaerobic bacteria and genital-mycoplasmas (e.g., U. urealyticum, and M. hominis) with the use of the methods previously reported [6, 7]. We centrifuged and stored the remaining-fluid in polypropylene-tubes at -70°C. We measured MMP-8 concentrations in stored-AF as previously reported [9, 10]. The sensitivity of the test was <0.3 ng/mL. Both intra- and inter-assay coefficients of variation were <10%. AF inflammation was defined as an elevated AF MMP-8 concentration (>23 ng/mL) as previously reported [9]. AF was regarded as sterile if the AF culture was negative and there was no AF inflammation (i.e., AF MMP-8 levels were <23 ng/mL).

### Statistical analysis

The comparisons of continuous variables for clinical characteristics and pregnancy outcomes were performed using Kruskal-Wallis test and the comparisons of proportions were made using Pearson's chi-square test. Spearman rank correlation test was used to investigate the relationship between amniocentesis-to-delivery interval and inflammatory status in the extra-placental membranes (i.e., inflammation-free extra-placental membranes, chorio-deciduitis only, and chorio-deciduitis with amnionitis). Mann-Whitney U test was used for the comparison of amniocentesis-to-delivery interval between each inflammatory status group in extra-placental membranes. The linear-by-linear association test was used to investigate for trend. Logistic regression analysis was used to examine the relationship between chorio-deciduitis with or without amnionitis, and amniocentesis-to-delivery interval controlling for the effect of any other potential confounding variables. Data were analyzed using SPSS Statistics 20.0. Statistical significance was defined as P < .05.

## Results

The clinical characteristics and pregnancy outcomes in cases with PTL and in those with preterm-PROM are shown in Table 1 and Table 2, respectively. There was a significant difference in GA at amniocentesis, GA at delivery, vaginal delivery or Cesarean section after trial of labor, and antenatal corticosteroids use between the groups according to amniocentesis-to-delivery interval in both PTL and preterm-PROM (P<0.05 for each, Table 1 and Table 2). There was also a significant difference in antenatal use of antibiotics between the three amniocentesis-to-delivery interval groups in preterm-PROM, but not PTL (see Table 1 and Table 2).

**Table 1:** Clinical characteristics and pregnancy outcomes according to amniocentesis-to-delivery interval (i.e., ≤2 days, 2–7 days, and >7 days) in 133 patients with preterm labor and intact membranes (PTL) and sterile amniotic fluid.

	≤2 days	2–7 days	>7 days	P^†^
	n = 77, 57.9%	n = 17, 12.8%	n = 39, 29.3%	
Maternal age, years (mean csSD)	30.6 nal ag29.3 nal ag	30.3 nal agNS		
Parity (age,69% (53/77)	41% (7/17)	41% (16/39)	< .01	
GA at amniocentesis, weeks (mean ± SD)	33.5 amnio33.1 amnioc	31.5 amnioce < .001		
Cervical dilatation at amniocentesis,	1 [0, 6]	1 [0, 3]	1 [0, 4]	NS
cm (median, range) ^m^				
GA at delivery, weeks (mean ± SD)	33.5 deliv33.6 deliv	34.8 deliver < .05		
Vaginal delivery or Cesarean section	36% (28/77)	65% (11/17)	64% (25/39)	< .01
after trial of labor				
Clinical chorioamnionitis	6% (5/77)	0% (0/17)	0% (0/39)	NS
Birth weight, g (mean tisean2202.4 eight, g2297.7 eight, g	2344.9 ight, g (NS			
Male gender of newborn	58% (45/77)	47% (8/17)	49% (19/39)	NS
1 min Apgar score < 7	42% (32/77)	35% (6/17)	26% (10/39)	NS
5 min Apgar score < 7	23% (18/77)	18% (3/17)	10% (4/39)	NS
Umbilical cord arterial pH at birth ≤mbili^∫^	7% (5/67)	9% (1/11)	4% (1/24)	NS
Antenatal use of antibiotics	21% (16/77)	29% (5/17)	23% (9/39)	NS
Antenatal corticosteroids	35% (27/77)	35% (6/17)	59% (23/39)	< .05
Tocolytics	74% (57/77)	88% (15/17)	90% (35/39)	NS

^†^ Among three groups, Kruskal-Wallis test was used for the comparisons of continuous variables and Pearson's chi-square test was used for the comparisons of proportions.

^∫^ Of 133 cases, 102 cases were only included in the analysis, because the test of umbilical arterial pH was not performed in 31 cases due to the failure in extraction of umbilical arterial blood at birth.

^‡^ Of 133 patients, the information about cervical-dilatation at amniocentesis was not available in 2 cases.

*SD*, standard deviation; *GA*, gestational age; *NS*, not significant.

**Table 2:** Clinical characteristics and pregnancy outcomes according to amniocentesis-to-delivery interval (i.e., ≤2 days, 2–7 days, and >7 days) in 156 patients with preterm premature rupture of membranes (preterm-PROM) and sterile amniotic fluid.

	≤2 days	2–7 days	>7 days	P^†^
	n = 73, 46.8%	n = 54, 34.6%	n = 29, 18.6%	
Maternal age, years (mean cst bi29.5 ± 4.6	30.9 ± 4.2	30.8 ± 5.2	NS	
Parity (≥1)	45% (33/73)	44% (24/54)	45% (13/29)	NS
GA at amniocentesis, weeks (mean ± SD)	34.8 ± 1.2	33.4 ± 1.8	30.9 ± 2.8	< .001
Cervical dilatation at amniocentesis,	1 [0, 4]	1 [0, 3]	0 [0, 3]	NS
cm (median, range)				
GA at delivery, weeks (mean ± SD)	34.9 ± 1.2	34.1 ± 1.8	33.7 ± 2.2	< .005
Vaginal delivery or Cesarean section	59% (43/73)	78% (42/54)	79% (23/29)	< .05
after trial of labor				
Clinical chorioamnionitis	1% (1/73)	2% (1/54)	0% (0/29)	NS
Birth weight, g (mean tisea^††^	2499.5 ± 388.8	2237.0 ± 442.4	2129.6 ± 490.4	< .005
Male gender of newborn^††^	54% (39/73)	76% (41/54)	59% (17/29)	< .05
1 min Apgar score < 7	26% (19/73)	41% (22/54)	31% (9/29)	NS
5 min Apgar score < 7	10% (7/73)	11% (6/54)	14% (4/29)	NS
Umbilical cord arterial pH at birth ≤ 7.15^∫^	2% (1/62)	6% (3/48)	0% (0/25)	NS
Antenatal use of antibiotics^‡^	73% (51/70)	94% (50/53)	90% (26/29)	< .005
Antenatal corticosteroids^‡‡^	22% (16/72)	67% (36/54)	83% (24/29)	< .001
Tocolytics^‡‡‡^	22% (15/69)	53% (28/53)	45% (13/29)	< .005

^†^ Among three groups, Kruskal-Wallis test was used for the comparisons of continuous variables and Pearson's chi-square test was used for the comparisons of proportions.

^††^ Of 156 cases, the data about birth weight and male gender of newborn was not available in 1 case.

^∫^ Of 156 cases, 135 cases were only included in the analysis, because the test of umbilical arterial pH was not performed in 21 cases due to the failure in extraction of umbilical arterial blood at birth.

^‡^ Of 156 cases, the data about antenatal use of antibiotics was not available in 4 cases.

^‡‡^ Of 156 cases, the data about antenatal corticosteroids use was not available in 1 case.

^‡‡‡^ Of 156 cases, the data about tocolytics use was not available in 5 cases.

*SD*, standard deviation; *GA*, gestational age; *NS*, not significant.

**Fig 1:**
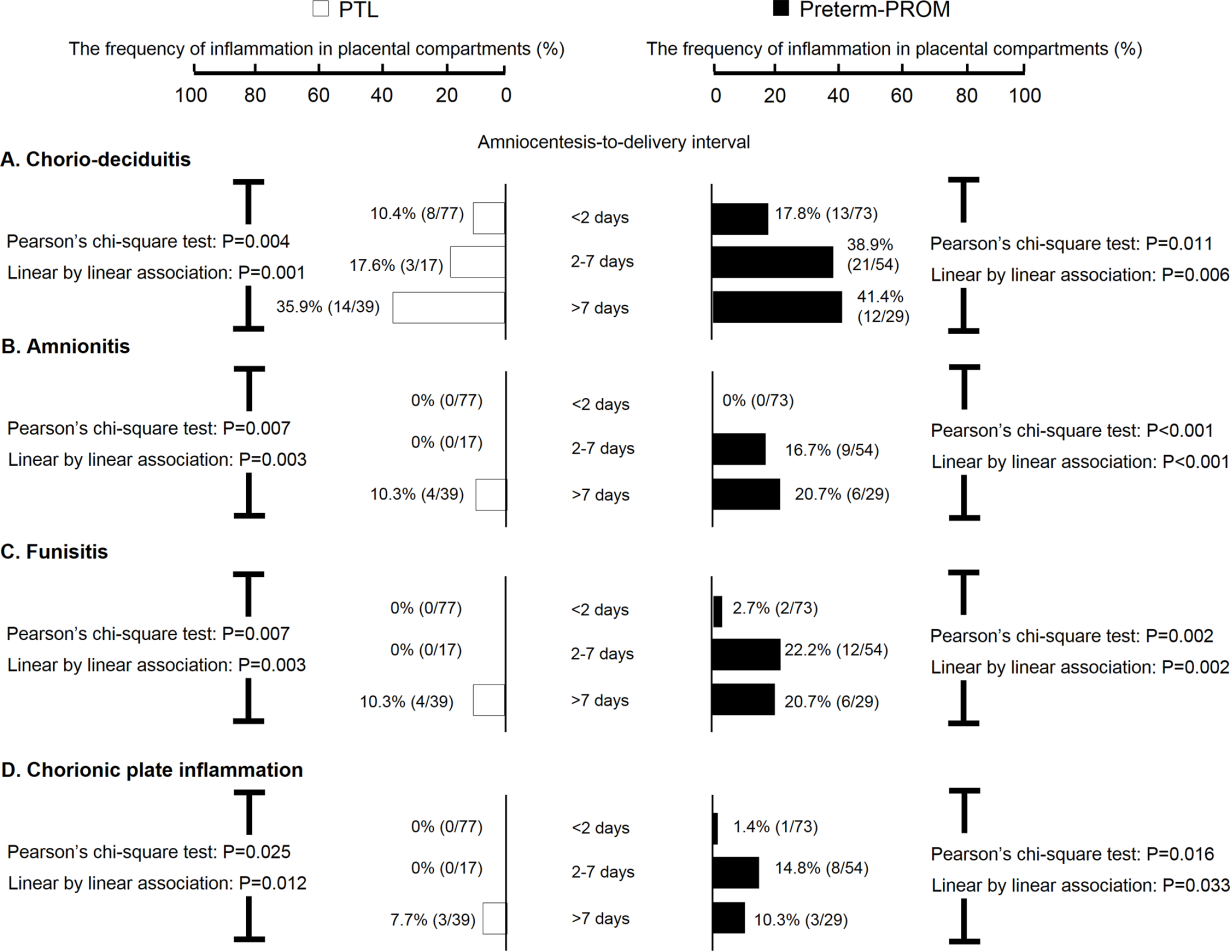
Frequency of inflammation in placental compartments according to amniocentesis-to-delivery interval. The frequency of chorio-deciduitis (A), amnionitis (B), funisitis (C), and chorionic plate inflammation (D) according to amniocentesis-to-delivery interval (i.e., ≤2 days, 2–7 days, and >7 days) is shown in patients with PTL and preterm-PROM. Frequency and P-values are shown.

**Fig 2:**
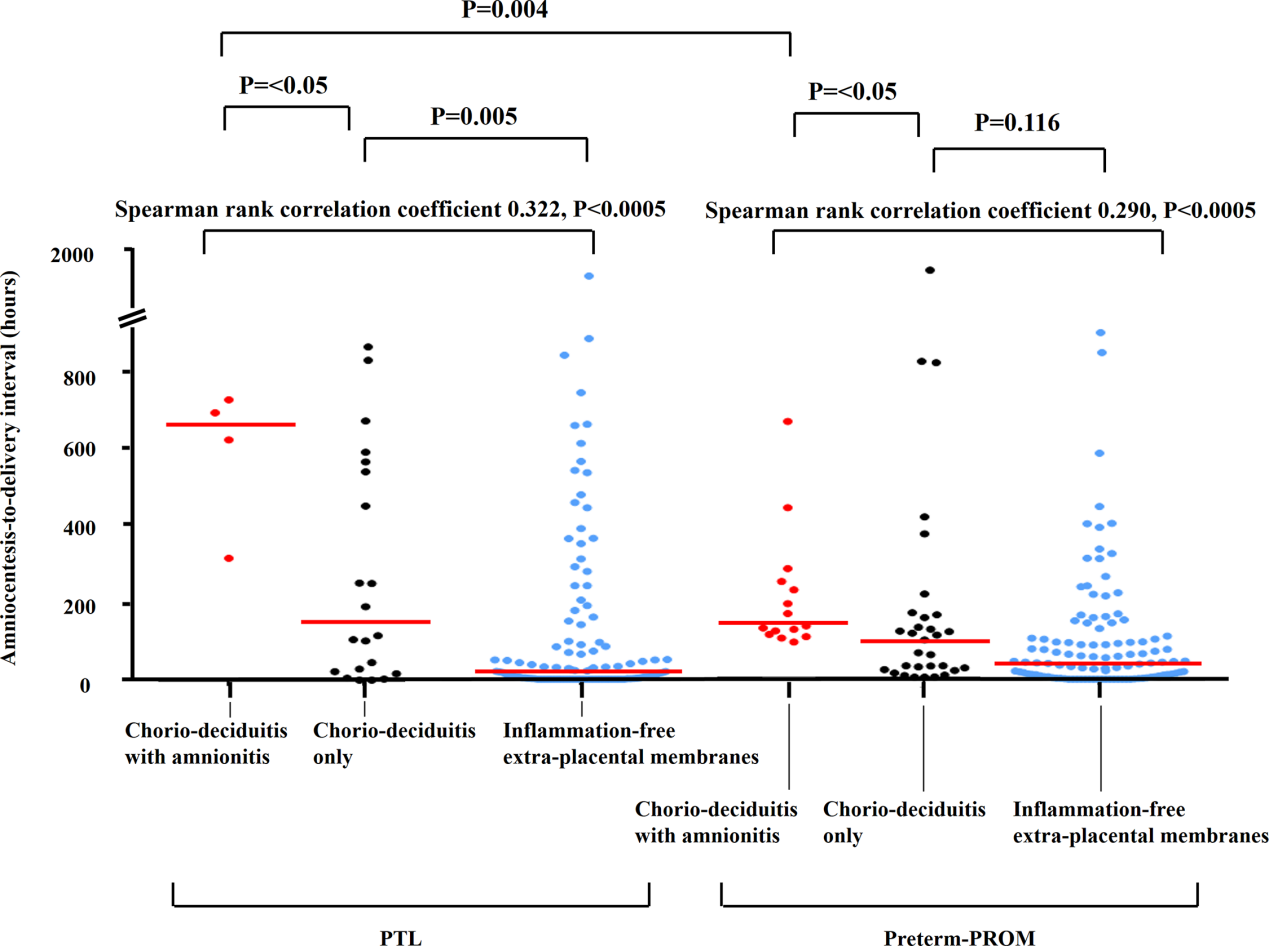
Amniocentesis-to-delivery interval in accordance with histologic status of the extra-placental membranes. Amniocentesis-to-delivery interval is shown according to histologic status of the extra-placental membranes (i.e., inflammation-free, chorio-deciduitis only, and chorio-deciduitis with amnionitis) in patients with PTL (median, range; 20.6 hours [0.01–1794.8 hours] vs. 113.9 hours [0.10–1093.1 hours] vs. 654.5 hours [312.2–718.2 hours]) and preterm-PROM (median, range; 42.9 hours [0.01–1267.2 hours] vs. 95.2 hours [0.01–1833.9 hours] vs. 131.3 hours [90.2–655.0 hours]). P-values and Spearman rank correlation coefficients are shown.

Inflammation in each of the placental compartments (i.e., chorio-deciduitis, amnionitis, funisitis, and chorionic plate inflammation) increased with increasing amniocentesis-to-delivery interval in both PTL and preterm-PROM (P<0.05 for each using linear-by- linear association analysis; Fig 1). Chorio-deciduitis developed within 2 days of the documentation of sterile AF in 10.4% (8/77) cases of PTL and 17.8% (13/73) cases of preterm-PROM (Fig 1A). However, amnionitis did not appear until 2 days of the documentation of sterile AF in the preterm-PROM cohort and until 7 days in the PTL cohort (Fig 1B). Amnionitis was first documented on day 6 after documentation of sterile AF in the preterm-PROM cohort and on day 14 in the PTL cohort (Fig 2). Of note, histologic amnionitis was never present without chorio-deciduitis in both PTL and preterm-PROM cohorts (data not shown).

Fig 2 shows amniocentesis-to-delivery interval according to the inflammatory status in the extra-placental membranes. There was a strong correlation between amniocentesis-to-delivery interval and inflammation in the extra-placental membranes in both PTL (r = 0.322; P<0.0005; Spearman rank correlation test) and preterm-PROM (r = 0.290; P<0.0005; Spearman rank correlation test). Moreover, median [interquartile range (IQR)] amniocentesis-to-delivery interval was longer in cases of chorio-deciduitis with amnionitis than in cases of chorio-deciduitis only in both PTL (645.4 [319.5] vs. 113.9 [526.9] hours, P<0.05) and preterm-PROM (131.3 [135.4] vs. 95.2 [140.5] hours, P<0.05) (Fig 2). Of note, chorio-deciuditis with amnionitis developed later in PTL than in preterm-PROM (P<0.005) (Fig 2).

**Fig 3:**
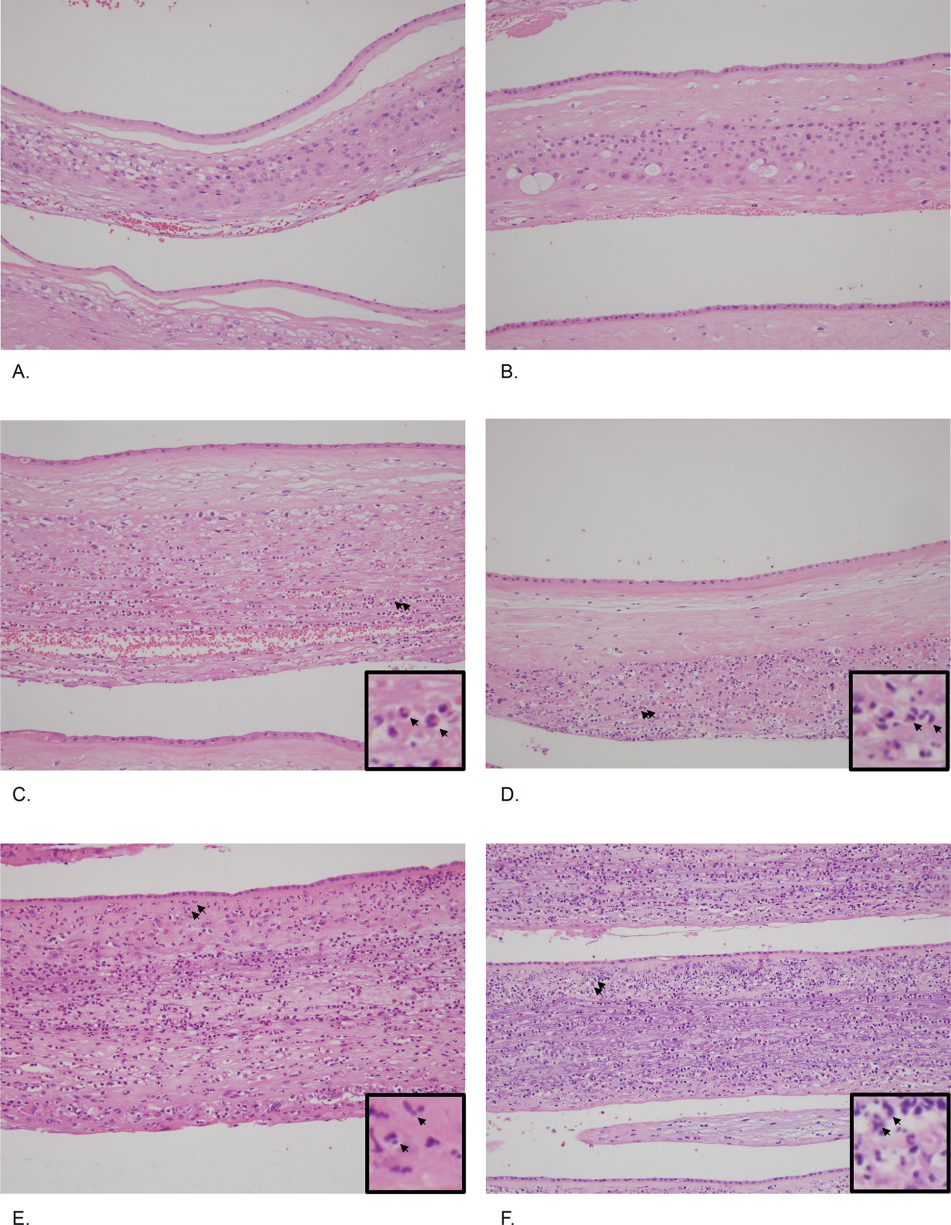
Histopathology of extra-placental membranes (chorio-decidua and amnion) (These images are based on the magnification setting X200). Hematoxylin and eosin stained histologic sections of extra-placental membranes (chorio-decidua and amnion) are shown for inflammation-free placenta (A, PTL; and B, preterm-PROM), choriodeciduitis only (C, PTL; and D, preterm-PROM) and chorio-deciduitis with amnionitis (E, PTL; and F, preterm-PROM). Neutrophils in the chorio-decidua are indicated with arrows in panel C and panel D (see insets of panels C-D), and arrows in panel E and panel F indicate neutrophilic infiltration in amnion (see insets of panels E-F).

Fig 3 shows the histopathology of extra-placental membranes (chorio-decidua and amnion) in patients with PTL (panels A, C and E) and preterm-PROM (panels B, D and F). Hematoxylin and eosin stained histologic sections of extra-placental membranes (chorio-decidua and amnion) are shown for inflammation-free placenta (A, PTL; and B, preterm-PROM), choriodeciduitis only (C, PTL; and D, preterm-PROM) and chorio-deciduitis with amnionitis (E, PTL; and F, preterm-PROM). Neutrophils are only shown in the chorio-decidua in panel C and panel D of Fig 3, and neutrophilic infiltration extends into the amnion in panel E and panel F of Fig 3.

To determine the relative value of clinical parameters in the prediction of chorio-deciduitis with or without amnionitis, we conducted multiple logistic regression analysis with variables commonly regarded as confounding factors for the development of chorio-deciduitis and amnionitis. Of all these independent variables, amniocentesis-to-delivery interval retained statistical significance in the prediction of both chorio-deciduitis and amnionitis after adjusting for confounding variables such as GA at delivery in PTL (Table 3) while early GA at delivery was an independent risk factor for amnionitis after adjusting for the contribution of potential confounding variables in preterm-PROM (Table 4).

**Table 3:** Relationship of various independent variables with the development of chorio-deciduitis and amnionitis analyzed by overall logistic regression analysis in PTL with sterile amniotic fluid.

Prediction of chorio-deciduitis	Odds ratio	95% CI	p value
Amniocentesis-to-delivery interval	1.002	1.001–1.004	0.021
Gestational age at delivery	0.800	0.628–1.019	0.071
Antibiotics use	0.260	0.080–0.851	0.026
Antenatal corticosteroids use	0.724	0.229–2.289	NS
Cervix dilatation at amniocentesis	0.746	0.448–1.243	NS
Vaginal delivery or Cesarean section	6.284	1.665–23.720	0.007
after trial of labor			
Clinical chorioamnionitis	0.261	0.020–3.360	NS
5 min Apgar score < 7	0.497	0.103–2.394	NS
Prediction of amnionitis			
Amniocentesis-to-delivery interval	1.003	1.000–1.005	0.048
Gestational age at delivery	1.000	0.638–1.568	NS
Antibiotics use	0.241	0.019–3.048	NS
Antenatal corticosteroids use	0.343	0.026–4.429	NS
Cervix dilatation at amniocentesis	1.015	0.392–2.625	NS
Vaginal delivery or Cesarean section	3.443	0.228–51.996	NS
after trial of labor			
Clinical chorioamnionitis	31485802.54	0.000-	NS
5 min Apgar score < 7	0.232	0.012–4.637	NS

*PTL*, preterm labor and intact membranes; *CI*, confidence interval.

**Table 4:** Relationship of various independent variables with the development of chorio-deciduitis and amnionitis analyzed by overall logistic regression analysis in preterm-PROM with sterile amniotic fluid.

Prediction of chorio-deciduitis	Odds ratio	95% CI	p value
Amniocentesis-to-delivery interval	1.001	1.000–1.003	0.123
Gestational age at delivery	0.791	0.613–1.020	0.071
Antibiotics use	1.877	0.620–5.682	NS
Antenatal corticosteroids use	0.611	0.253–1.475	NS
Cervix dilatation at amniocentesis	1.012	0.667–1.537	NS
Vaginal delivery or Cesarean section	2.606	0.987–6.884	0.053
after trial of labor			
Clinical chorioamnionitis	0.001	0.000-	NS
5 min Apgar score < 7	0.581	0.162–2.088	NS
Prediction of Amnionitis			
Amniocentesis-to-delivery interval	1.002	0.999–1.004	0.176
Gestational age at delivery	0.677	0.488–0.940	0.020
Antibiotics use	0.001	0.000-	NS
Antenatal corticosteroids use	1.244	0.328–4.721	NS
Cervix dilatation at amniocentesis	1.179	0.621–2.239	NS
Vaginal delivery or Cesarean section	2.860	0.516–15.839	NS
after trial of labor			
Clinical chorioamnionitis	0.117	0.003–4.395	NS
5 min Apgar score < 7	1.050	0.149–7.417	NS

*preterm-PROM*, preterm premature rupture of membranes; *CI*, confidence interval.

## Discussion

Principal findings of this study are that, in cases with confirmed sterile AF, longer time is required to develop histologic chorio-deciduitis with amnionitis than chorio-deciduitis alone in the setting of both PTL and preterm-PROM and prolongation of pregnancy is an independent predictor of both chorio-deciduitis and amnionitis in PTL.

The chorio-decidua, the outermost layer of the extra-placental membranes, is exposed to the vagina via the cervical canal and may be readily accessible by ascending intra-uterine infection. In contrast, the amnion, the innermost layer of the extra-placental membranes, is more protected and is not readily accessible by ascending intra-uterine infection [1–3]. We hypothesize, therefore, that more time is required for ascending intra-uterine infection to cause chorio-deciduitis with amnionitis than chorio-deciduitis alone. Unfortunately, no study has yet been reported in humans to test this hypothesis, and there are no good animal models of ascending infection in the setting of PTL or preterm-PROM. To test this hypothesis, we therefore chose a large cohort of women with PTL or preterm-PROM, who underwent amniocentesis to exclude intra-uterine infection, and who subsequently delivered preterm and had their placentas examined by placental pathologists. In order to avoid the possibility of trans-placental infection and to enrich the population with cases that were likely to have a prolonged amniocentesis-to-delivery interval, we chose to exclude patients who had preexisting intra-amniotic infection and/or inflammation (as evidenced by a positive AF culture or elevated AF MMP-8 concentration).

Our data demonstrated a strong correlation between amniocentesis-to-delivery interval and inflammatory status in the extra-placental membranes (Fig 2). Moreover, the median amniocentesis-to-delivery interval was longer in cases of chorio-deciduitis with amnionitis than in cases of chorio-deciduitis alone in both the PTL and preterm-PROM cohorts (Fig 2). It should be noted that, while histologic chorio-deciduitis was evident as early as one day after documentation of sterile AF in both the PTL and preterm-PROM groups, histologic evidence of amnionitis first appeared on day 6 in the preterm-PROM cohort and day 14 in the PTL cohort (Fig 2).

In this study, amnionitis developed later in the PTL group than in the preterm-PROM group (Fig 2). Given that the amnion lines the amniotic cavity, in patients with PTL, microorganisms or neutrophils originating within the maternal decidua must migrate through the chorion and chorio-amniotic interface before reaching the amnion [1–3]. However, in the context of ruptured membranes, microorganisms from the vagina may directly invade the amnion by entering the intra-uterine cavity through site of membranes rupture [1–3]. This may explain why PTL is likely to need more time to manifest amnionitis than preterm-PROM.

There is no information about the relationship between prolongation of pregnancy and the development of acute histologic chorioamnionitis in cases of PTL. Moreover, most data about the latency after preterm-PROM analyzed clinical, but not histologic, chorioamnionitis [11–17], and very little data regarding the length of the latency period and histologic chorio-deciduitis / amnionitis in the setting of preterm-PROM is controversial [18–20]. While some studies have reported an association between the development of acute chorioamnionitis and prolonged rupture of membranes [18, 19], other study has not [20] (see Table 5). However, all of these studies have one major limitation, which is that they failed to perform amniocentesis and confirm the absence of pre-existing intra-amniotic infection/inflammation, thereby failing to exclude a major source of bias leading to a shorter amniocentesis-to-delivery interval in cases of intra-amniotic infection/inflammation [9, 21].

**Table 5:** Relationship between the latency after ROM and chorioamnionitis in previous studies.

Primary author (year)	n	GA	GA	Inclusion of	CA as a main outcome	Does CA increase with the latency
[Reference number]		at delivery	at ROM	AF study	(histologic or clinical)	between ROM and delivery?
Cox et al. (1995) [11]	129	NA	30–34 weeks	Not included	Clinical	Yes
Naef et al. (1998) [12]	120	NA	34–37 weeks	Not included	Clinical	Yes
Ramsey et al. (2005) [13]	430	<37 weeks	>24 weeks	Not included	Clinical	Yes
Aziz et al. (2008) [14]	1,168	24–34 weeks	24–34 weeks	Not included	Clinical	No
Nayot et al. (2008) [15]	1,535	NA	25–37 weeks	Not included	Clinical	Yes
Ekin et al. (2014) [16]	204	NA	24–34 weeks	Not included	Clinical	Yes
Mercer et al. (1993) [17]	93	NA	32–37 weeks	Not included	Clinical	Yes
McElrath et al. (2003) [18]	430	<28 weeks	<28 weeks	Not included	Histologic	Yes
Üstün et al. (1998) [19]	61	>37 weeks	>37 weeks	Not included	Histologic	Yes
Ghidini et al. (1998) [20]	191	22–32 weeks	22–32 weeks	Not included	Histologic	No

*GA*, gestational age; *NA*, not available; *ROM*, rupture of membranes; *AF*, amniotic fluid; *CA*, chorioamnionitis.

Our results also show that prolongation of pregnancy is an independent predictor of both chorio-deciduitis and amnionitis after adjusting for confounding variables (i.e., GA at delivery) in patients with PTL (Table 3). These results are consistent with that of Lee and colleagues’ [22] who reported that a longer duration of labor was associated with a higher frequency of acute-chorioamnionitis and funisitis in pregnant women in labor at term with intact membranes, although it should be noted that this study did not include women with PTL and there was no information on infection or inflammation in the AF. How then is PTL with sterile AF causally related to histologic chorio-deciduitis with increasing amniocentesis-to-delivery interval? It has been proposed that, in addition to causing the cervix to efface and dilate, uterine contractions have a “suction-like effect” that draws vaginal fluid with a microbial load into the uterine cavity [23, 24]. Moreover, vaginal delivery or Cesarean section after trial of labor was an independent risk factor for the development of chorio-deciduitis in patients with PTL (Table 3). It is plausible that delivery mode influence the histologic finding of the extra-placental membranes because the patient who delivered vaginally or tried to deliver vaginally experienced more times of uterine contractions and probably received more times of digital examinations. Indeed, this result is similar to the previous report that acute-histologic chorioamnionitis was significantly more common in women with regular uterine contractions with cervical dilatation ≥2cm than in those without regular uterine contraction [25].

The major strengths of the study are that it included a large cohort of singleton preterm pregnancies with AF biomarkers and placental histology (n = 289) and that we specifically excluded patients with preexisting intra-amniotic infection/inflammation, which is known to have a strong effect on both the amniocentesis-to-delivery interval and the incidence of acute chorioamnionitis [5, 9, 21, 26]. The potential weaknesses of this study are as follows. Firstly, we measured latency as amniocentesis-to-delivery interval, since it is difficult to define the precise time at which PTL or preterm-PROM developed. However, we do not believe this is a major source of bias, since our prior study has shown that there is no relationship between intra-amniotic inflammation and the time elapsed between membranes rupture and amniocentesis after the first 6 hours of membranes rupture [27]. Secondly, we used “the absence of pre-existing intra-amniotic inflammation” instead of “the absence of pre-existing uterine cervicitis” as a starting point of ascending intra-uterine infection, although the first step of ascending intra-uterine infection is the bacterial infection to the uterine cervix in theory. However, all the previous studies about the uterine cervicitis (defined as an elevated cervical fluid inflammatory cytokine concentration) and acute histologic chorioamnionitis consistently reported that the presence or absence of uterine cervicitis did not discriminate between the presence and absence of acute histologic chorioamnionitis [28–31]. Indeed, we found “the absence of intra-amniotic inflammation”(defined as AF MMP-8 < 23 ng/ml) is superior to “the absence of uterine cervicitis” (defined as cervical fluid IL-6 < 350 pg/ml) in the identification of inflammation-free placenta in another retrospective cohort of our database which consisted of 38 singleton pregnant women who underwent amniocentesis and cervical fluid collection before 37 weeks of gestation and delivered within 7 days of amniocentesis, and we could predict “inflammation-free placenta” only with the use of “the absence of intra-amniotic inflammation” (sensitivity, 89.5% (17/19); specificity, 57.9% (11/19); positive predictive value, 68.0% (17/25); negative predictive value, 84.6% (11/13); positive likelihood ratio [95% confidence interval], 2.1250 [1.2268, 3.6808], negative likelihood ratio [95% confidence interval], 0.1818 [0.0464, 0.7126]), but not “the absence of uterine cervicitis” (sensitivity, 68.4% (13/19); specificity, 52.6% (10/19); positive predictive value, 59.1% (13/22); negative predictive value, 62.5% (10/16); positive likelihood ratio [95% confidence interval], 1.444 [0.8219, 2.5386], negative likelihood ratio [95% confidence interval], 0.6000 [0.2730, 1.3186]) (unpublished data). Thirdly, we regarded “5 min Apgar score < 7” instead of umbilical cord arterial pH at birth and abnormal fetal heart rate pattern (FHR) as the representative of fetal distress in the overall logistic regression analysis for the relationship between various independent variables and the development of chorio-deciduitis and amnionitis, although the Apgar scoring system remains as relevant for the prediction of neonatal survival in preterm and term neonates as it was almost 50 years ago [32, 33] and the validity of the relationship between certain FHR patterns (i.e., degree of FHR variability and depth of decelerations) and fetal academia and/or 5-minute Apgar score ≥7 was demonstrated [34]. However, we did not have umbilical cord arterial pH result at birth in 31 patients (23.3%) among 133 cases with PTL and in 21 patients (13.5%) among 156 cases with preterm-PROM due to the failure in the extraction of umbilical cord arterial blood at birth. Moreover, it would seem that it is inadequate to accept abnormal FHR pattern as an objective indicator of fetal distress because there is high inter-observer and intra-observer variability in the interpretation of FHR tracings [35–37]. Finally, we did not quantitatively evaluate the frequency of uterine contraction as an independent risk factor of chorio-amnionitis. However, it would seem that we should consider the amplitude or intensity in addition to the frequency of PTL for the exact quantitation of uterine contractility. Unfortunately, external uterine tocodynamometry is unlikely to represent the exact intensity of uterine contraction. Indeed, Paul MJ et al. demonstrated that external tocodynamometry reliably distinguishes between uterine contractions and uterine quiescence in preterm pregnancies but does not adequately measure the intensity of contractions [38]. However, we did not routinely apply the intrauterine pressure monitor for the exact measurement of the intensity of uterine contractions in patients with PTL, and moreover, we did not find any study reporting the quantitation of internal uterine activity with the use of intrauterine pressure monitor as an objective quantitation method of uterine contractility in patients with PTL. For these reasons, we did not consider the markers related to the measurement of uterine contraction as an independent risk factor of chorio-amnionitis. However, we already performed the overall logistic regression analysis for the relationship between various independent variables and the development of chorio-deciduitis and amnionitis separating patients with PTL from those with preterm-PROM, and moreover, we already included “cervix dilatation at amniocentesis” in the overall logistic regression analysis, because cervix dilatation was likely to represent the result of uterine contractions and a previous report demonstrated that the more advanced the cervical dilatation, the higher the risk of acute-histologic chorioamnionitis [25].

To our knowledge, this is the first study in humans showing that more time is required to develop histologic chorio-deciduitis with amnionitis than chorio-deciduitis alone in the setting of both PTL and preterm-PROM after documentation of sterile AF. We also show that prolongation of pregnancy is an independent predictor of both chorio-deciduitis and amnionitis in the setting of PTL. Further research is needed to better understand the spatio-temporal migration of ascending intra-uterine infection in the extra-placental membranes, and to determine the effect of pregnancy prolongation on neonatal outcome.
